# Adiposity, diabetes, lifestyle factors and risk of gastroesophageal reflux disease: a Mendelian randomization study

**DOI:** 10.1007/s10654-022-00842-z

**Published:** 2022-02-04

**Authors:** Shuai Yuan, Susanna C. Larsson

**Affiliations:** 1 Unit of Cardiovascular and Nutritional Epidemiology, Institute of Environmental Medicine, Karolinska Institutet, Nobels väg 13, 17177 Stockholm, Sweden; 2 Unit of Medical Epidemiology, Department of Surgical Sciences, Uppsala University, Uppsala, Sweden

**Keywords:** Coffee, Diabetes, Gastroesophageal reflux disease, Mendelian randomization, Obesity, Smoking

## Abstract

Adiposity, diabetes, and lifestyle factors are linked to gastroesophageal reflux disease (GERD) in observational studies. We conducted a two-sample Mendelian randomization analysis to determine whether those associations are causal. Independent genetic variants associated with body mass index (BMI), waist circumference (with and without adjustment for BMI), type 2 diabetes, smoking, and alcohol, coffee and caffeine consumption at the genome-wide significance level were selected as instrumental variables. Summary-level data for GERD were available from a genome-wide association meta-analysis of 71,522 GERD cases and 261,079 controls of European descent from the UK Biobank and QSkin Sun and Health studies. The odds ratio (OR) of GERD was 1.49 (95% confidence interval (CI), 1.40–1.60) for one standard deviation (SD) increase in BMI, 1.07 (95% CI, 1.04–1.10) for one-unit increase in log-transformed OR of type 2 diabetes, and 1.41 (95% CI, 1.31–1.52) for one SD increase in prevalence of smoking initiation. There were suggestive associations with GERD for higher genetically predicted waist circumference (OR per one SD increase, 1.14, 95% CI, 1.02–1.26) and caffeine consumption (OR per 80 mg increase, 1.08, 95% CI, 1.02–1.15). Genetically predicted waist circumference adjusted for BMI, alcohol or coffee consumption was not associated GERD. This study suggests causal roles of adiposity, diabetes, and smoking, and a possible role of high caffeine consumption in the development of GERD.

## Introduction

Gastroesophageal reflux disease (GERD) is a common gastrointestinal disorder, affecting approximately 13% of the worldwide population and 20% of the adult population in the western countries [1, 2]. GERD impairs the patients’ life quality and increases the risk of other esophageal complications, such as esophagitis, esophageal strictures, Barrett esophagus, and esophageal adenocarcinoma [1, 2]. Epidemiological studies have revealed several possible risk factors for GERD, including excess adiposity [3–7], diabetes [8], smoking [6, 7, 9–12], alcohol consumption [11, 13], and coffee and caffeine consumption [6, 14]. However, evidence on most associations is equivocal with inconsistent findings across studies [15–19]. Additionally, unobserved confounding, misclassification, reverse causality, and other biases may hinder causal inference in these associations in observational studies. Determining the causal link of potentially modifiable risk factors with GERD is of great importance in understanding the etiology of this disease as well as in preventing and managing the disease in the clinical settings.

Mendelian randomization (MR) design uses genetic variants as instrumental variables for an exposure and can strengthen the causal inference [20]. This design can diminish residual confounding since the genetic variants are randomly assorted at conception and therefore have limited correlations with environmental and self-adopted factors [20]. In addition, the MR design can minimize the possibility of reverse causality because genetic variants cannot be modified by the development or progression of the disease [20]. A recent MR study in the UK Biobank study found an association for waist-to-hip ratio, but not for body mass index (BMI), smoking or caffeine consumption with risk of GERD, whereas the analyses for smoking and caffeinated-coffee consumption were underpowered [21]. Here, we conducted a two-sample MR study to examine the associations of overall and central adiposity, diabetes, smoking and alcohol, coffee, and caffeine consumption with risk of GERD based on more GERD cases and genetic instruments that explain more phenotypic variances.

## Methods

### Study design

**Fig. 1:**
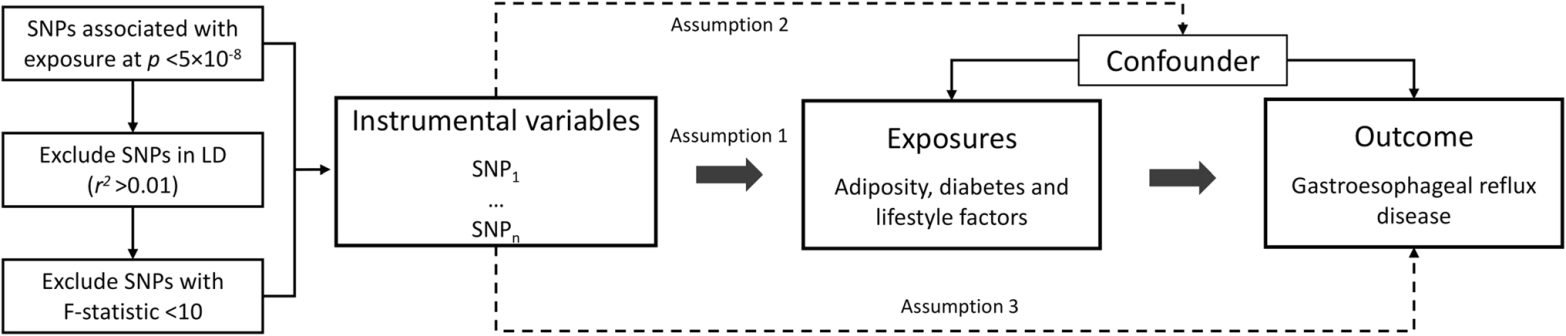
Study design overview. LD, linkage disequilibrium; SNPs, single-nucleotide polymorphisms. There are three assumptions of Mendelian randomization design. The first assumption is that the genetic variants used as instrumental variables should be robustly associated with the exposure; the second assumption is that the used genetic variants should not be associated with any confounders; and the third assumption is that the selected genetic variants should affect the risk of the outcome merely through the risk factor, not via alternative pathways

Figure 1 shows the study design overview. This study was based on summary-level data on measures of adiposity, type 2 diabetes, lifestyle factors, and GERD from published genome-wide association studies (GWASs) and consortia. All studies included in the cited GWASs and consortia had been approved by a relevant review board and involved participants had given informed consent. The present MR analyses were approved by the Swedish Ethical Review Authority (2019‐02,793).

### Instrument variable selection

Genetic variants (i.e., single nucleotide polymorphisms, SNPs) associated with BMI [22], waist circumference [23], type 2 diabetes [24], smoking initiation [25], and alcohol [25], coffee [26], and caffeine [27, 28] consumption at the genome-wide significance level (*p* < 5 × 10^–8^) were obtained from corresponding GWASs (Table 1). Smoking initiation was defined as a binary phenotype representing whether an individual had ever smoked cigarettes regularly (current or past smoker) [25]. SNPs associated with waist circumference adjusted for BMI was used to examine the BMI-independent effect of waist circumference [23]. Linkage disequilibrium among the SNPs was estimated using 1000 Genomes European panel as the reference population. Independent SNPs (i.e., SNPs without linkage disequilibrium, defined by *r*^*2*^ <0.001 and clumping window > 10,000 kb) were used as instrumental variables.

**Table 1:** Information on used studies and consortia

Exposure or outcome	Unit	Participants included in analysis	Adjustments	Identified SNPs	Variance explained	F-statistic	OR at 80% power	PubMed ID
Body mass index	SD (~ 4.8 kg/m^2^)	806 834 European-descent individuals	Age, sex, and genetic 1–5 principal components	312	7.7%	286	≤ 0.95 or ≥ 1.05	25,673,413
Waist circumference	SD	224 459 European-descent individuals	Age and study-specific covariates	47	1.2%	86	≤ 0.89 or ≥ 1.11	25,673,412
Waist circumference adjusted for BMI	SD	224 459 European-descent individuals	Age, body mass index and study-specific covariates	70	1.5%	72	≤ 0.90 or ≥ 1.10	25,673,412
Type 2 diabetes	One-unit in log-transformed odds ratio	228 499 type 2 diabetes cases and 1 178 783 non-cases of multi-ancestries	Age, sex, and the first ten genetic principal components	558	19.0%	140	≤ 0.97 or ≥ 1.03	32,541,925
Smoking initiation	SD in prevalence of smoking initiation	1 232 091 European-descent individuals	Age, sex, and the first ten genetic principal components	378	2.3%	21	≤ 0.92 or ≥ 1.08	30,643,251
Alcohol drinking	SD increase of log-transformed alcoholic drinks/week	941 280 European-descent individuals	Age, sex, and the first ten genetic principal components	99	0.7%	24	≤ 0.86 or ≥ 1.15	30,643,251
Coffee consumption	50% change	375 833 European-descent individuals	Age, sex, body mass index, total energy, proportion of typical food intake and 20 genetic principal components	14	0.5%	119	≤ 0.84 or ≥ 1.18	31,046,077
Caffeine consumption	80 mg (close to caffeine from one cup of coffee)	9876 European-descent individuals	Age, sex, study-site, fasting status, family structure, genetic principal components, and smoking status	2	1.3%	2190	≤ 0.89 or ≥ 1.11	27,702,941
GERD	Log-transformed odds ratio	71 522 GERD cases and 261 079 controls of European descent	Recruitment age, genetic sex, the first ten principal components, and cryptic relatedness	–	–	–	–	31,527,586

*GERD* Gastroesophageal reflux disease; *ID* Identifier; *OR* Odds ratio; *SD* Standard deviation; *SNPs* Single-nucleotide polymorphism.

### Gastroesophageal reflux disease data source

Summary-level data on the associations of exposure-related SNPs with GERD were obtained from a genome-wide association meta-analysis of the UK Biobank study and QSkin Sun and Health Study including a total of 71 522 GERD cases and 261 079 controls of European descent [29]. GERD cases were defined by filed codes of self-report, International Classification of Disease 9 and 10, the Office of Population Censuses and Surveys, and treatment/medicine in the UK Biobank study (68 535 cases and 250 910 controls), and self-reported heartburn and medical records of reflux medications in QSkin Sun and Health Study (2987 cases and 10 169 controls) [29]. The GWAS analysis was adjusted for recruitment age, genetic sex, the first ten principal components, and cryptic relatedness.

### Statistical analysis

The inverse variance weighted method was used as the main statistical method (the random-effects model for the exposure constructed by ≥ 3 SNPs and the fixed-effect model for the exposure constructed by < 3 SNPs). Several sensitivity analyses, including the weighted median [30], MR-Egger [31], MR-PRESSO [32], and contamination mixture method [33] were conducted to examine the consistency of associations and detect and correct for horizontal pleiotropy. Assuming > 50% of weight from valid SNPs, the weighted median method can provide consistent estimates [30]. MR-Egger analysis can generate pleiotropy-corrected estimates if the intercept test detects significant horizontal pleiotropy (*p* for intercept < 0.05); however, the model is usually underpowered [31]. MR-PRESSO method can detect outlying SNPs and provide estimates after removal of outliers [32]. The embedded distortion test can examine the difference in estimates before and after removing outliers [32]. The contamination mixture method can provide robust estimates in analysis using hundreds of SNPs as instrumental variables with the presence of invalid SNPs [33]. To assess whether genetic liability to type 2 diabetes is associated with GERD risk independently of BMI, we performed the multivariable MR analysis with adjustment for genetically predicted BMI. Cochrane’s Q was used to assess the heterogeneity of estimates of SNPs. Power was estimated using an online tool (Table 1) [34]. Associations with *p* value < 0.007 (0.05/7 exposures) were deemed significant associations, and associations with a *p* value ≥ 0.007 and ≤ 0.05 were regarded as suggestive associations. All tests were two-sided and performed using the TwoSampleMR [35], MR-PRESSO [32] and MendelianRandomization [36] packages in the R software (version 4.0.2).

## Results

**Fig. 2:**
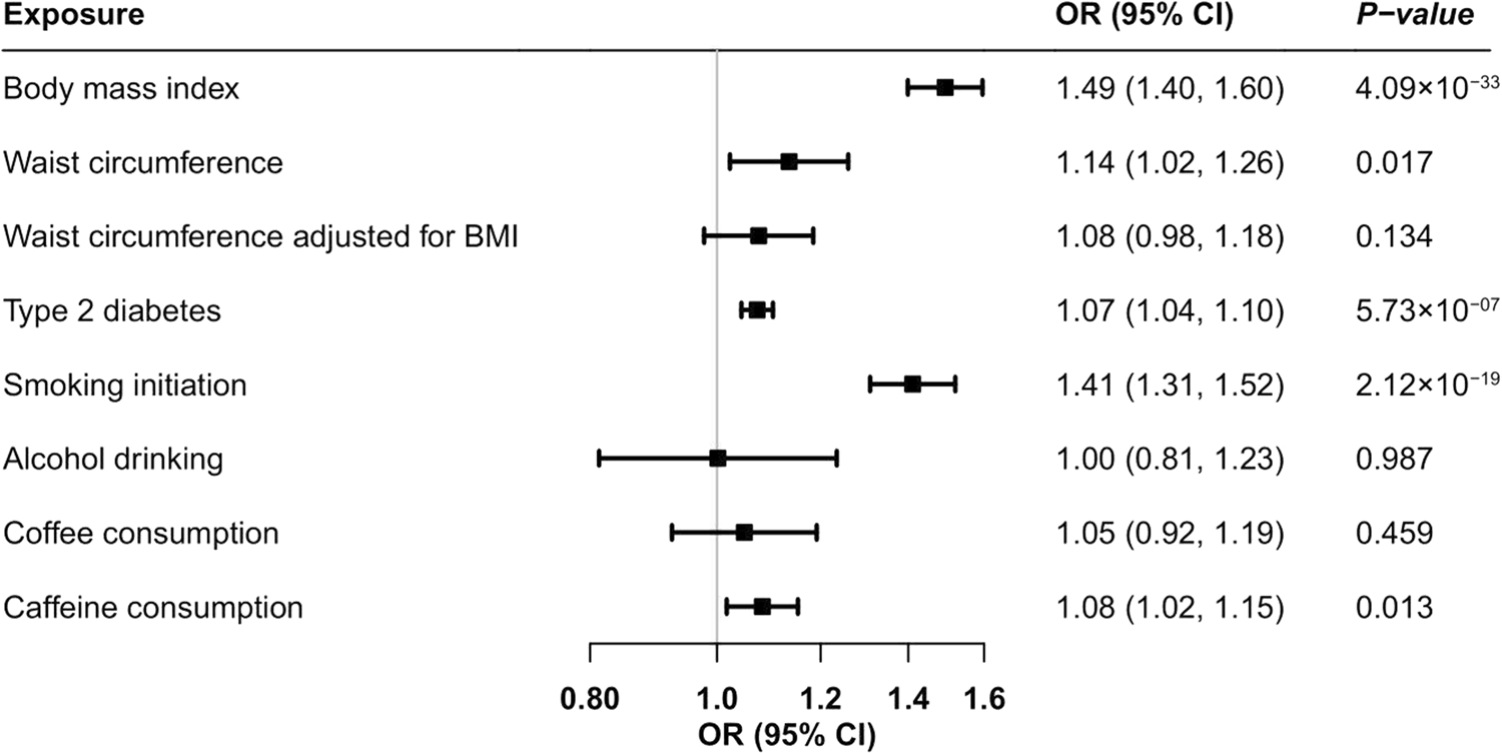
Associations of genetically proxied adiposity, type 2 diabetes, and lifestyle factors with risk of gastroesophageal reflux disease. BMI, body mass index; CI, confidence interval; OR, odds ratio. Estimates were obtained from the inverse variance weighted method with random-effects with the except for estimate for caffeine consumption that was obtained from the inverse variance weighted method with fixed-effects.

Higher genetically predicted BMI and genetic predisposition to type 2 diabetes (not diabetes diagnosis) and smoking initiation was associated with an increased risk of GERD (Fig. 2). The odds ratio (OR) of GERD was 1.49 (95% confidence interval (CI), 1.40, 1.60; *p* = 4.09 × 10^–33^) for one standard deviation (SD) increase in BMI, 1.07 (95% CI, 1.04, 1.10; *p* = 5.73 × 10^–7^) for one-unit increase in log-transformed OR of type 2 diabetes (not diabetes diagnosis), and 1.41 (95% CI, 1.31, 1.52; *p* = 2.12 × 10^–19^) for one SD increase in prevalence of smoking initiation. The association for type 2 diabetes slightly attenuated but remained significant in the multivariable MR analysis with adjustment for genetically predicted BMI (OR, 1.05, 95% CI, 1.03, 1.08; *p* = 2.76 × 10^–4^). There were suggestive associations for genetically predicted waist circumference (OR per one SD increase, 1.14, 95% CI, 1.02, 1.26; *p* = 0.017) and caffeine consumption (OR per 80 mg increase, 1.08, 95% CI, 1.02, 1.15; *p* = 0.013) (Fig. 2). The association for waist circumference attenuated in the analysis using SNP associated with waist circumference adjusted for BMI (OR per one SD increase, 1.08, 95% CI, 0.98, 1.18; *p* = 0.134) (Fig. 2). We did not observe any association of genetically predicted alcohol or coffee consumption with risk of GERD in the main analysis (Fig. 2).

Associations for all exposures remained overall consistent in sensitivity analyses (Table 2). The association became stronger for waist circumference adjusted for BMI in MR-Egger analysis and for waist circumference in the contamination mixture analysis (Table 2). Horizontal pleiotropy was observed in the MR-Egger analysis of BMI and type 2 diabetes (*p* for intercept < 0.05) (Table 2). One to ten outliers were detected by MR-PRESSO analyses; however, the associations remained consistent after removal of these outliers and no difference in estimates before and after removing outliers was observed (*p* for distortion test > 0.05) (Table 2).

**Table 2:** Associations of genetically predicted risk factors with gastroesophageal reflux disease in Mendelian randomization sensitivity analyses

Exposure	Used SNPs	Cochrane’s Q	Weighted median			MR-Egger		
			OR	95% CI	*P*	OR	95% CI	*P*
Body mass index	312	781	1.37	1.27, 1.48	6.50 × 10^–15^	1.05	0.90, 1.23	0.529
Waist circumference	38	81	1.06	0.95, 1.20	0.307	0.98	0.75, 1.29	0.906
Waist circumference adjusted for BMI	62	144	1.09	0.99, 1.20	0.082	1.47	1.03, 2.10	0.041
Type 2 diabetes	278	770	1.03	1.00, 1.07	0.055	0.98	0.93, 1.03	0.425
Smoking initiation	202	546	1.33	1.23, 1.45	6.27 × 10^–12^	1.12	0.83, 1.51	0.454
Alcohol drinking	71	210	0.98	0.78, 1.22	0.839	0.92	0.61, 1.37	0.676
Coffee consumption	11	21	1.07	0.95, 1.20	0.256	1.23	0.99, 1.53	0.100

Exposure	P_pleiotropy_^a^	P_distortion test_^b^	MR-PRESSO^c^			Contamination mixture		
			OR	95% CI	*P*	OR	95% CI	*P*
Body mass index	2.57 × 10^–6^	0.847	1.48	1.39, 1.58	2.64 × 10^–29^	1.62	1.51, 1.72	1.65 × 10^–35^
Waist circumference	0.265	0.795	1.15	1.05, 1.26	0.006	1.14	1.05, 1.26	0.003
Waist circumference adjusted for BMI	0.086	0.744	1.07	0.99, 1.15	0.115	1.05	0.98, 1.12	0.134
Type 2 diabetes	6.52 × 10^–5^	0.216	1.06	1.03, 1.09	6.84 × 10^–6^	1.05	1.03, 1.07	1.80 × 10^–7^
Smoking initiation	0.121	0.293	1.47	1.37, 1.58	5.33 × 10^–22^	1.57	1.43, 1.67	2.62 × 10^–25^
Alcohol drinking	0.618	0.652	1.09	0.92, 1.30	0.310	1.13	0.90, 1.84	0.137
Coffee consumption	0.131	0.077	0.98	0.85, 1.14	0.814	1.09	1.00, 1.20	0.052

*BMI* Body mass index; *CI* Confidence interval; *NA* Not available; *OR* Odds ratio; *SNPs* Single-nucleotide polymorphisms. Sensitivity analyses could not be performed for caffeine consumption due to few SNPs (< 3 SNPs)

^a^*P* values for pleiotropy were p values for MR-Egger intercept test and a *p* value < 0.05 indicates a statistically significant pleiotropic effect

^b^*P* values for distortion were obtained from MR-PRESSO test and a *p* value < 0.05 indicates a statistically significant difference between estimates before and after outlier removal. *P* of distortion test was not available for the analysis of coffee consumption due to no outlier detected

^c^There were 6 outliers detected in MR-PRESSO analysis of BMI, 2 in waist circumference, 4 in waist circumference adjusted for BMI, 10 in type 2 diabetes, 7 in smoking initiation, 4 in alcohol drinking, and 1 in coffee consumption

## Discussion

This MR study found that higher genetically predicted BMI and genetic liability to type 2 diabetes and smoking were associated with increased GERD risk. There were suggestive associations of genetically predicted higher waist circumference and caffeine consumption with an increased risk of GERD. The association for waist circumference attenuated after adjustment for genetically predicted BMI. Limited data was observed in support of an association of genetically predicted alcohol and coffee consumption with GERD.

Review articles of the association between obesity and GERD have found consistent evidence that overweight and obesity were associated with an ascended risk of GERD [3, 4, 17]. In a meta-analysis of 20 observational studies (mostly case–control and cross-sectional studies) with a total of 18 346 GERD patients, overweight and obesity was associated with a 57% and 115% higher risk of GERD, respectively [3], which is in line with our findings. This association is also supported by a cohort study including 29 610 Norwegians where weight loss was found to be associated with an increased odds of loss of GERD [5] and several randomized controlled trials [7]. However, a recent MR study of adiposity-related phenotypes in relation to GERD found that central adiposity, measured by waist-hip ratio instead of overall adiposity measured by BMI, showed a causal association with the increased risk of GERD [21]. Even though our study confirmed the positive association between central obesity measured by waist circumference and GERD, the association attenuated after adjustment for BMI. Thus, our data supported a stronger impact of overall adiposity, measured by BMI, compared to central adiposity on GERD. Several underlying mechanisms may explain the association between obesity and GERD, including esophageal motor abnormalities, low esophageal sphincter abnormalities, elevated intra-abdominal and intragastric pressures, increased frequency of transient sphincter relaxation, and esophageal inflammation [4, 37].

Gastrointestinal symptoms are frequently encountered in patients with type 2 diabetes. The association between type 2 diabetes and GERD was assessed in a meta-analysis including nine observational studies and the OR of GERD was 1.61 for diabetic individuals compared to non-diabetic controls [8]. Our MR study strengthened the causal nature of this positive association and further revealed that this association was independent of BMI. However, our MR estimate could not be compared with observational estimates since the risk of GERD was scaled to modelled liability to diabetes instead of diabetes diagnosis. Even though pathological pathways linking diabetes to GERD have not been fully investigated, the adverse effects of its upstream factors (e.g., obesity, smoking, etc.) on GERD and the autonomic neuropathy, especially vagal nerve damage in diabetic patients may explain this association [8, 38].

Evidence in support of an association between smoking and GERD was generally consistent [6, 7, 9–12]. A clinical trial in 14 smokers found that resuming smoking habits after abstaining from smoking for 48 h greatly increased acid reflux and heartburn [12]. The positive association between smoking and GERD was also revealed in case–control studies [11]. In The Trøndelag Health Study, smoking cessation was associated with an improvement in severe GERD in individuals with normal weight and anti-reflux medication, but not in individuals with minor GERD symptoms, overweight, or those using anti-reflux medication less than weekly [9]. Another study confirmed this association by comparing GERD improvement between the group that successful stopped smoking by varenicline and the group that failed to stop smoking [10]. Our MR study based on genetic data further strengthened the positive association between smoking and GERD even though we could not assess the interaction effects of body weight and the severity of the disease. A recent MR study did not observe this association, which might be caused by inadequate power [21]. Several mechanisms may decipher the increased risk of GERD in smokers, including reduced lower esophageal sphincter resting pressure (blocked cholinergic receptors by nicotine) and prolonged acid clearance time caused by reduced salivary secretion rate and bicarbonate concentration [39].

Consumptions of alcohol and coffee, two major beverages, have been associated with GERD risk with inconsistent or weak evidence for alcohol [11, 13, 15] and coffee consumption [6, 14, 15, 19]. A meta-analysis including 29 studies found an increased risk of GERD in regular alcohol drinkers compared to non- or occasional drinkers; however, this finding was majorly based on cross-sectional and case–control studies and therefore were prone to residual confounding from other alcohol intake-correlated lifestyles and behaviors as well as misclassification bias [13]. Our MR study did not observe a positive association between alcohol consumption and GERD risk, although we could not rule out the possibility that the observed null finding was led by an inadequate power or a possible association of heavy alcohol consumption or alcohol abuse with GERD. With regard to coffee consumption, our finding was in line with some [19] but not all [14] previous studies. Likewise, our null finding on coffee consumption is risked by an inadequate power. In a prospective analysis of data from Nurses’ Health Study II, consumption of coffee, tea, and soda (all contain caffeine) was associated with an increased risk of GERD symptoms, which may partly support the observed positive for caffeine consumption [6, 14], although the associations for coffee, tea, and soda in that study did not vary by caffeine status. Given that limited data examined the link from overall caffeine consumption to GERD risk, more study is needed.

Limitations need considerations when interpreting our results. The important limitation is the possible effect of horizontal pleiotropy. In this study, we observed significant pleiotropic effects in the analyses of BMI and type 2 diabetes, but not for other exposures. However, the associations for BMI and diabetes were consistent in sensitivity analyses. Anti-diabetes medications may be a pleiotropic source in the analysis of type 2 diabetes [40]. From the time logic (medications after disease onset), this is a vertical pleiotropy, which does not influence causal inference. Even though our analyses were based on a GERD dataset with a large sample size, we might overlook weak associations, especially for the exposure constructed by few SNPs that explain a small phenotypic variance. We had inadequate power in the analyses for waist circumference adjusted for BMI, and in the analyses of alcohol and coffee consumption. Our analysis included approximately 30% of GERD cases defined by self-reported information only in the UK Biobank, which might introduce outcome misclassification. However, the GERD genome-wide association study found strong genetic correlations across three GERD phenotypes defined by ICD10, self-reported GERD, and use of GERD medication, respectively (0.92 < *r*_*g*_ < 0.97) [29], which indicated a good validity of self-report outcome data. Thus, the bias caused by self-report data should be minimal. Our study was confined to individuals of European descent, which reduced the population structure bias. On the other hand, this confinement may limit the generalizability of our findings to other populations. For smoking behaviors, the interaction effect with body weight and the severity of GERD could not be examined in this study based on summary-level data. For alcohol and coffee consumption, the effects of different types of alcohol or coffee could not be differentiated.

In summary, this MR study suggests causal roles of obesity, diabetes, and smoking in the development of GERD. The possible association between high caffeine consumption and an increased risk of GERD warrants confirmations.

## Data Availability

The datasets analyzed in this study are publicly available summary statistics. Data used can be obtained upon a reasonable request to the corresponding author.
